# Comparing Connective Tissue Grafts and Collagen Matrix in Modified Coronally Advanced Tunnel Technique for RT1 Gingival Recession: A Randomized Controlled Clinical Trial

**DOI:** 10.1186/s12903-025-06259-w

**Published:** 2025-06-03

**Authors:** Ahmed Elbana, Wafaa Saleh, Jilan Youssef

**Affiliations:** 1 Periodontology, Diagnosis, and Oral Radiology Department, Faculty of Dentistry, Mansoura University, Mansoura, Egypt; 2 Mansoura City, Mansoura, 33516 Egypt

**Keywords:** Modified coronally advanced tunnel technique, Connective tissue graft, Collagen matrix, Gingival recession

## Abstract

**Background:**

The effectiveness of the modified coronally advanced tunnel (MCAT) technique in treating isolated gingival recession Type 1 (RT1) has been evaluated in a few studies. Hence, this prospective randomized controlled clinical trial was directed to assess the MCAT technique with connective tissue graft (CTG) or collagen matrix (CM) in improving clinical outcomes for isolated gingival recession type 1 (RT1), mainly the attached gingiva width (AGW) as a primary outcome. Despite the clinical relevance of AGW, there is a gap in the literature with limited studies concerning it as a primary outcome.

**Methods:**

Forty patients were chosen and randomly assigned into either the control group (MCAT and CTG) or the test group (MCAT and CM) for short-term assessment (6 months) of single (RT1) gingival recession treatment. The primary outcomes were attached gingiva width (AGW), recession depth (RD), recession width (RW), and mean root coverage% (MRC%). The secondary outcomes included gingival thickness (GT), keratinized tissue width (KTW), periodontal probing depth (PPD), clinical attachment level (CAL), plaque index (PI), gingival index (GI), pink esthetic score (PES), root coverage esthetic score (RES), and patient-reported outcome measures (PROMs). The chi-square (χ²) test, Mann-Whitney U test, t-test, and Friedman test were used for statistical analysis of the outcomes.

**Results:**

Forty patients aged 18 to 45 years were enrolled in the current study. Evaluation of the primary outcomes (AGW, RD, RW) showed significant differences in the studied groups from the baseline to the final follow-up period (p-value ≤ 0.05). The MRC % results showed significant improvement after the 6-month follow-up period. For the control group, the MRC% was 97.08 ± 9.09, while for the test group, the MRC% was 96.75 ± 7.99. The PI, GI, PPD, CAL, KTW, PES, or RES values did not significantly differ among the two groups from the baseline to the final follow-up period.

**Conclusion:**

Our study supported the efficacy of the MCAT in isolated gingival recession coverage. The CM may act as an alternative to CTG by increasing the patient’s satisfaction and reducing tissue morbidity and surgical time.

**Trial registration:**

The current clinical trial was retrospectively registered in ClinicalTrials.gov (ID: NCT06065774) and released on 11/18/2024.

## Introduction

Gingival recession is a common periodontal condition of apical migration of the free gingival margin and progressive tissue loss. It compromises the aesthetic appearance of the patients through exposure of the root surfaces besides its impact on increased tooth sensitivity, root caries, and periodontal health [1]. 

The objectives of the treatment are to achieve root coverage with long-term periodontal stability, restore esthetics and function, and improve the patient’s quality of life [2]. The current literature discussed the minimal required dimensions of keratinized tissue around teeth. It has been suggested that a 1.5 to 2 mm width of keratinized gingiva was required for periodontal stability. Therefore, there is a consensus that a thin gingival phenotype is a diseased condition and requires intervention to achieve long-term predictability [3]. Thus, the goal of gingival recession treatment is expanded to augment and stabilize the keratinized attached tissue with proper dimensions [4]. 

A new perspective in the attached gingiva (AG) was introduced by Tarnow et al., who stated that there is a critical distinction between the zones of the AG and keratinized gingiva (KG). The AG zone is extended from the end of the sulcus to the mucogingival junction (MGJ), which is significantly different from the KG zone, which is extended from the free gingival margin to the MGJ [5]. The clinical significance of this differentiation becomes critical in cases where only the keratinized gingiva exists without attachment to the underlying tissues. Therefore, getting an attached connective tissue covering the root surface becomes an additional important target of the treatment to establish stable predictable periodontal outcomes. This ends the myth of KTW and focuses on the reality of connective tissue attachment to the underlying bony and hard recipient bed [6, 7]. 

Various surgical techniques are used for gingival recession management, each of which has its rationale for selection. Common surgical techniques include the free gingival graft (FGG) [8] and the coronally advanced flap (CAF) used with and without the connective tissue graft (CTG) [9, 10]. In addition to grafting procedures, enamel matrix derivatives [11], platelet-rich fibrin, and other bioactive mediators such as recombinant human platelet-derived growth factors have been used with improved outcomes [12, 13]. 

The CAF has long been the standard technique for gingival recession treatment [9, 10]. From conventional to minimally invasive approaches, gingival recession treatment has been developed and various surgical modalities have been designed to enhance precision and minimize invasiveness, such as the double papilla technique [14], lateral positioned flap [15], and vestibular incision subperiosteal tunneling access (VISTA) technique [16]. Furthermore, tunneling techniques [17] such as the modified tunnel technique [18], lateral tunnel technique [19], and others. These minimally invasive approaches have been used to overcome the limitations of conventional surgical procedures of CAF and its related complex flap design. These approaches preserve the tissue architecture, reduce postoperative discomfort, and promote improved healing [20]. 

The modified coronally advanced tunnel (MCAT) technique has been used for gingival recession treatment, providing a promising outcome for different types of gingival recession defects. As the CTG is the benchmark for soft tissue grafting, the combination of MCAT and CTG was used, combining both benefits and giving a synergistic effect for recession coverage with predictable outcomes in terms of soft tissue augmentation and root coverage [21, 22]. 

Moreover, advances in soft tissue substitutes, such as collagen matrix (CM), to be used as an alternative to CTG made the surgical technique less invasive [23]. CM acts as a scaffold for mechanical support, cell attachment, and migration. It promotes blood clot stabilization for tissue ingrowth and proliferation. Various CMs of porcine or bovine origin have been investigated in combination with The MCAT to avoid traditional autogenous grafts and their associated palatal site morbidity, making the MCAT a less invasive and more patient-friendly technique [24, 25]. The improved clinical therapeutic outcomes of CM with the MCAT regarding root coverage and periodontal parameters (MRC%, GT, KTW) revolved considerable interest in CMs to investigate the evidence of their possibility of being considered as a substitute for CTGs for the treatment of soft tissue defects [26, 27]. 

The MCAT enhances the recession coverage outcomes with predictable clinical periodontal parameters. Recession depth (RD), recession width (RW), and mean root coverage percentage (MRC%) are all improved with the MCAT. Besides that gingival thickness (GT), keratinized tissue width (KTW) [28], Pink Esthetic Score (PES) [29], and Root Coverage Esthetic Score (RES) are also improved [30, 31]. Despite these positive indications, there is no randomized controlled clinical trial (RCT) that has studied the treatment of RT1 defects using MCAT with either CTG or CM focusing on all periodontal parameters besides the assessment of the attached gingiva width (AGW) after treatment. Therefore, our study aims to clinically evaluate the MCAT with either CTG or CM in Cairo class 1(RT1) isolated gingival recession focusing on AGW. The null hypothesis of the current study is that there are no significant changes between both treatment modalities in terms of primary and secondary outcomes.

## Materials and methods

Our RCT was designed as two-arm parallel groups. A total of forty participants were selected for inclusion in this study, all the cases had an isolated gingival recession classified under Cairo Class I (RT 1) [32]. The enrollment process involved a comprehensive discussion of all study procedures, and informed consent was signed by participants before inclusion. The protocol of our study followed the ethical standards of the Faculty of Dentistry, Mansoura University, Egypt, and was registered under reference number (A04060722). The study was registered on ClinicalTrials.gov (ID: NCT06065774) and released in 10-10-2023. The research spanned from July 2022 to February 2024 and was conducted in the Department of Oral Medicine and Periodontology, Faculty of Dentistry, Mansoura University, Egypt. The Consolidated Standards of Reporting Trials (CONSORT) guideline was the recommended standard used during writing the manuscript [32]. 

### Sample size calculation

The Difference in MRC% was used for sampling the study, based on a previous study that used the MCAT technique combined with either CTG or CM (90 ± 18% mm at control sites versus 71 ± 21% mm at test sites)( (*p* < 0.05) [21]., the sample size was detected based on an expected effect size of 1.42, Using version 3.1.9.4 of the G Power program representing this clinical parameter between the study groups observed as an improvement between 3 and 6 months of follow-up. Using a 2-tailed test with an α error set at 0.06 and 85.0% power, the sample size in each group was determined to ensure adequate statistical power. To account for variability, the standard deviation in the paired group was estimated to not exceed 30%. 18 subjects in each group were computed as a sample size, providing 80% power for a true difference of 20% between the groups. To consider the possibility of potential patient dropouts, 20 patients were ultimately recruited, protecting the study against unexpected patient dropouts to maintain its statistical power.

### Randomization and blinding

A randomization list was used, and sealed opaque envelopes were created by a coinvestigator (number 1) not involved in patient enrollment or the process of the study. These numbered envelopes (1 to 40) were gathered in a container. Another investigator (number 2) drew the envelope from the container to hand it to the surgeon to ensure proper patient enrollment. The envelope was opened just before the surgery by the surgeon.

#### A Coi

nvestigator (number 3) who was not involved in the treatment allocation measured the primary and secondary outcomes to ensure the assessor blinding. The MCAT procedure was used for both groups while the participants were informed about CTG harvesting in the control group before surgery.

### Eligibility criteria

The following criteria were included: individuals with age between 18 and 45 years, who were presented with isolated gingival recession (maxillary /or mandibular central incisors, lateral incisors, canines, and premolars) exceeding 2 mm in depth, without any interproximal attachment loss. Specifically, participants were selected to have no systemic disease, have less than ten cigarettes per day smoking; and with a full mouth plaque score of less than 15% [33]. 

Participants were excluded if they exhibited interproximal alveolar bone loss, were pregnant or lactating, heavy smokers; immunocompromised individuals, had prosthetics at the recession site, presented with cervical lesions such as abrasion, abfraction or caries, had an infection or any gingival lesion related to the area of gingival recession, or displayed poor oral hygiene.

### Intervention

For each participant surgical sites were meticulously evaluated, and strict aseptic measures were employed throughout all surgical procedures. Before the interventions, participants underwent professional nonsurgical periodontal therapy, and individualized instructions for oral cleaning were afforded one month before the surgical procedures. The randomization process, which was completed using a sealed opaque envelope, ensured an unbiased allocation of participants to either the MCAT technique with the CTG from the palate, which served as the control group, or using the CM (Mucoderm^®^Botiss) with the MCAT technique which served as the test group.

All surgical procedures for both groups were done by the same qualified, experienced surgeon (10 years experience). Following the protocol mentioned by Aroca et al. in 2013 [21], the MCAT technique was fulfilled as follows: after local anesthesia with 4% articaine, an incision through the gingival sulcus was performed using a microsurgical blade SM67. Specially designed tunneling knives with unique angles were utilized to avoid perforation of the tunnel. The interdental papilla adjacent to the defect was tunneled in a full-thickness approach, and it was delicately separated from the underlying tissues using mini curettes. The tunnel was thoroughly widened around the defect, creating a pouch for stabilization of the graft [34]. 

For the control group, palatal anesthesia was administered to harvest the palatal CTG. FGG was harvested then the epithelial layer was removed extra-orally using the 15c blade, this resulted in a high-quality graft of 1 to 1.5 mm thickness [35]. Following harvesting of the graft, it was placed in the prepared pouch over the biomodified root surface using 24% EDTA gel (2 min application, 2 min washing). The CTG was placed using positioning suturing and fixed in place inside the tunnel medially and distally using a fixation suture of a bioresorbable material (monocryl suture). Finally, the gingival tissues advanced coronally-repositioned securing the graft around the neck of the tooth at the level of the CEJ using sling sutures with 6/0 polypropylene sutures [21]. 

**Fig. 1:**
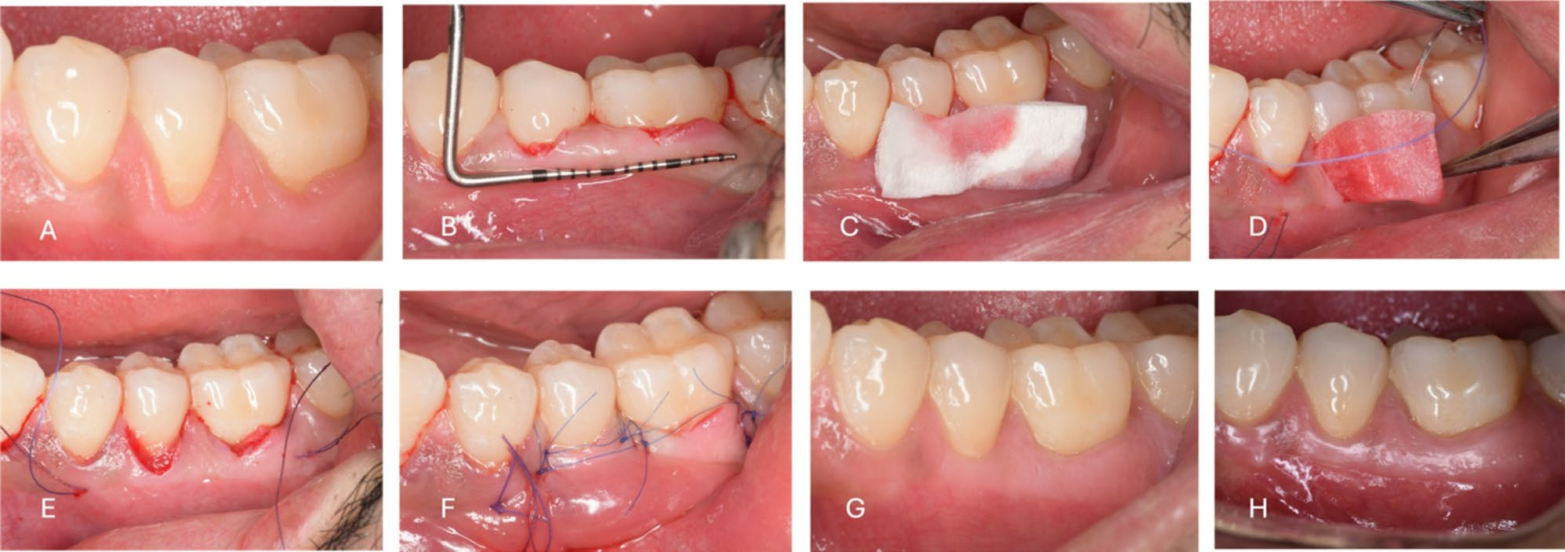
Modified coronally advanced tunnel technique (MCAT) with collagen matrix (CM) **(A)** Preoperative view of RT1 gingival recession at lower left premolar site **(B)** After preparation of the tunnel with specially designed tunneling instruments, the prepared tunnel was moved in a coronal direction in a tension free mobilization approach **(C)** Collagen matrix (CM) a xenogeneic dermal matrix of porcine origin (Mucograft^®^) was trimmed to match the dimension of the recession defect **(D)** CM was positioned through the tunnel using positioning suture of 6 − 0 monofilament resorbable suture **(E)** CM was sutured at the cementoenamel junction under the tunnel **(F)** The tunneled Flap is sutured coronally covering the recession defect and CM.**(G)** Clinical view at the 3-months postoperative, complete recession coverage is obvious (H) After 6- months of healing postoperatively a stable clinical outcome is still visible

**Fig. 2:**
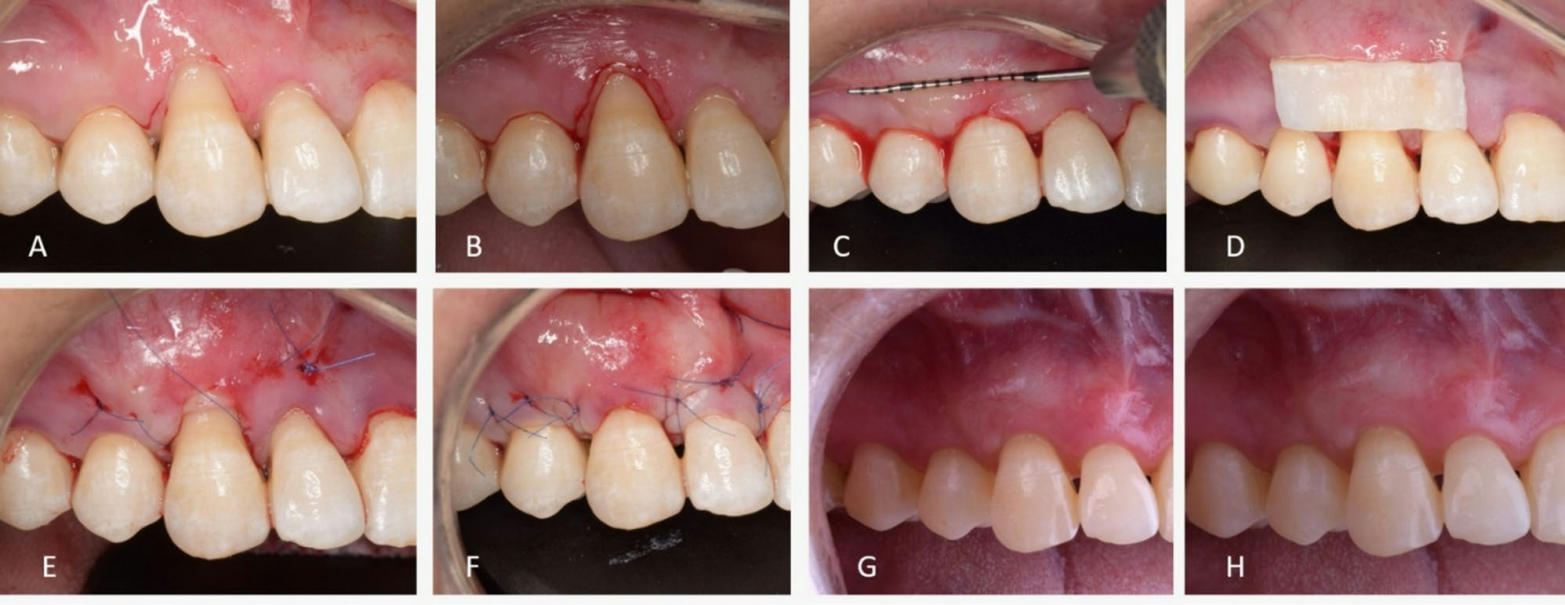
Modified coronally advanced tunnel technique (MCAT) with connective tissue graft (CTG) **(A)** Preoperative view of RT1 gingival recession at upper right canine **(B)** Gentle para marginal internal bevel incision using a micro-surgical blade to remove the sulcular epithelium and expose vascular connective tissue bed **(C)** After preparation of the tunnel with specially designed tunneling instruments, the prepared tunnel was moved in a coronal direction in a tension free mobilization approach **(D)** De-epithelialized CTG was harvested and trimmed to match the dimension of the recession defect **(E)** CTG was sutured at the cementoenamel junction under the tunnel **(F)** The tunneled Flap is sutured coronally covering the recession defect and CTG **(G)** Clinical view at the 3-months postoperative, complete recession coverage is obvious **(H)** After 6- months of healing postoperatively a stable clinical outcome is still visible

Alternatively, For the CM group, the collagen matrix was trimmed to an appropriate size, matching the dimensions of the recession defects, with an appropriate length and width of 10 × 5 mm. The matrix was then immersed in sterile saline to allow for expansion. Once fully saturated, the CM was positioned within the tunnel and secured using the same suturing technique used with the CTG [36]. (Figures 1 and 2)

### Postsurgical protocol

Following surgery, analgesics were prescribed twice daily for 5 days. Postoperative instructions were given, avoiding brushing for 14 days at the intervention site, and 0.1% chlorhexidine-based mouth rinse was used twice for 1 min daily for 2 weeks. Regular brushing resumed after the initial 14 days.

Seven days after the surgery the palatal sutures were taken out, while the removal of the sutures at the recipient site was postponed and detached between 14 and 21 days after the surgery. Recall appointments were arranged to assess the healing process and to motivate the patient to keep oral hygiene 1, 3, and 6 months postoperatively [22]. 

### Clinical assessments

Consistent evaluations of the involved teeth were executed at baseline, 3, and 6 months respectively. The clinical outcomes of the periodontal parameters were recorded by the same blinded investigator (number 3) throughout the study duration from the baseline to the final follow-up with a UNC 15 periodontal probe to maintain accuracy and reliability. All the clinical parameters were re-evaluated by the same investigator within 48-hour intervals for intra-examiner calibration.

Periodontal parameters were assessed, including the primary and the secondary outcomes. Recession depth (RD) is measured from the free gingival margin to the CEJ, recession width (RW) is measured horizontally at the level of CEJ, keratinized tissue width (KTW) is extended from the free gingival margin to the MGJ, gingival thickness (GT) is detected at the mid-buccal aspect of the involved tooth using the needle of the local anesthesia with a silicon stopper inserted till it stopped to avoid the tissue distortion that may occur after anesthesia infiltration, to ensure the accuracy of the measurements [37], AGW is calculated by deducting periodontal pocket depth (PPD) from KTW, and the clinical attachment level (CAL) is measured from the CEJ to the base of the sulcus.

The visual analog scale (VAS) at the baseline (the day after surgery), one week, and two weeks at the time of suture removal was used to report the degree of postoperative pain. Patients can mark what they feel on a 10 cm horizontal line from 0 = no pain to 10 = worst imaginable pain as their VAS pain score. The pink aesthetic score (PES) used a 14 score to assess the pink esthetic around the tooth. It is based on seven variables: mesial papilla, distal papilla, soft tissue level, soft tissue contour, alveolar process deficiency, soft tissue color, and texture. Each variable is assessed with a 2-1-0 score, with 2 being the best and 0 being the poorest score. The total PES is measured by adding the scores of all variables together. The root coverage aesthetic score (RES) was measured based on five variables on a 10-point scale: the gingival margin contour, soft tissue texture, MGJ alignment, and gingival color match, each variable is assessed with a 1 − 0 score according to whether it achieved an excellent outcome or not. Root coverage condition, either partially or completely is the determinant factor of the final score based on a 6-3-0 score, with 6 being complete root coverage, 3 being partial root coverage, and 0 being no root coverage achieved. The total RES is measured by adding the scores of all variables together. The patient-reported outcomes (PROMS) were measured using the visual analogue scale (VAS) on a 10-point scale. The patients were asked about their satisfaction with the treatment outcome using a yes or no question style; then they were asked to express the degree of their satisfaction on a scale from 0 to 10, with 0 meaning not satisfied at all and 10 meaning completely satisfied [29, 30]. 

#### Primary outcome variables

The primary outcome variables included: AGW, RD, RW, and MRC%. These variables centered on the assessment of the degree of root coverage and the improvement in attached gingiva from baseline, at the 3-month and 6-month marks.

#### Secondary outcome variables

The Secondary outcome variables comprised the evaluation of GT, KTW, PPD, CAL, PES, RES, PI, GI, and PROMS [38]. 

### Statistical analysis

Statistical Package for Social Sciences (SPSS) version 28 was the software for data analysis. Qualitative materials were presented in numbers and percentages. Kolmogorov- Smirnov test was used for quantitative data assessment. The data with normal distribution, mean and standard deviation were applied. Whereas for non-normality data, medians and ranges were applied. The chi-square test was used for categorical data (sex distribution), the Mann-Whitney U Test was used for non-normally distributed data, and the Friedman Test was used for repeated measures within the same group (VAS) [39]. 

The paired-difference t-test assessed the intergroup differences of the treatment for all numerical data such as AGW, RD, RW, GT, KTW, PPD, CAL, MRC%, PES, RES, and VAS. For. The P-value is statistically significant at ≤ 0.05.

## Results

**Fig. 3:**
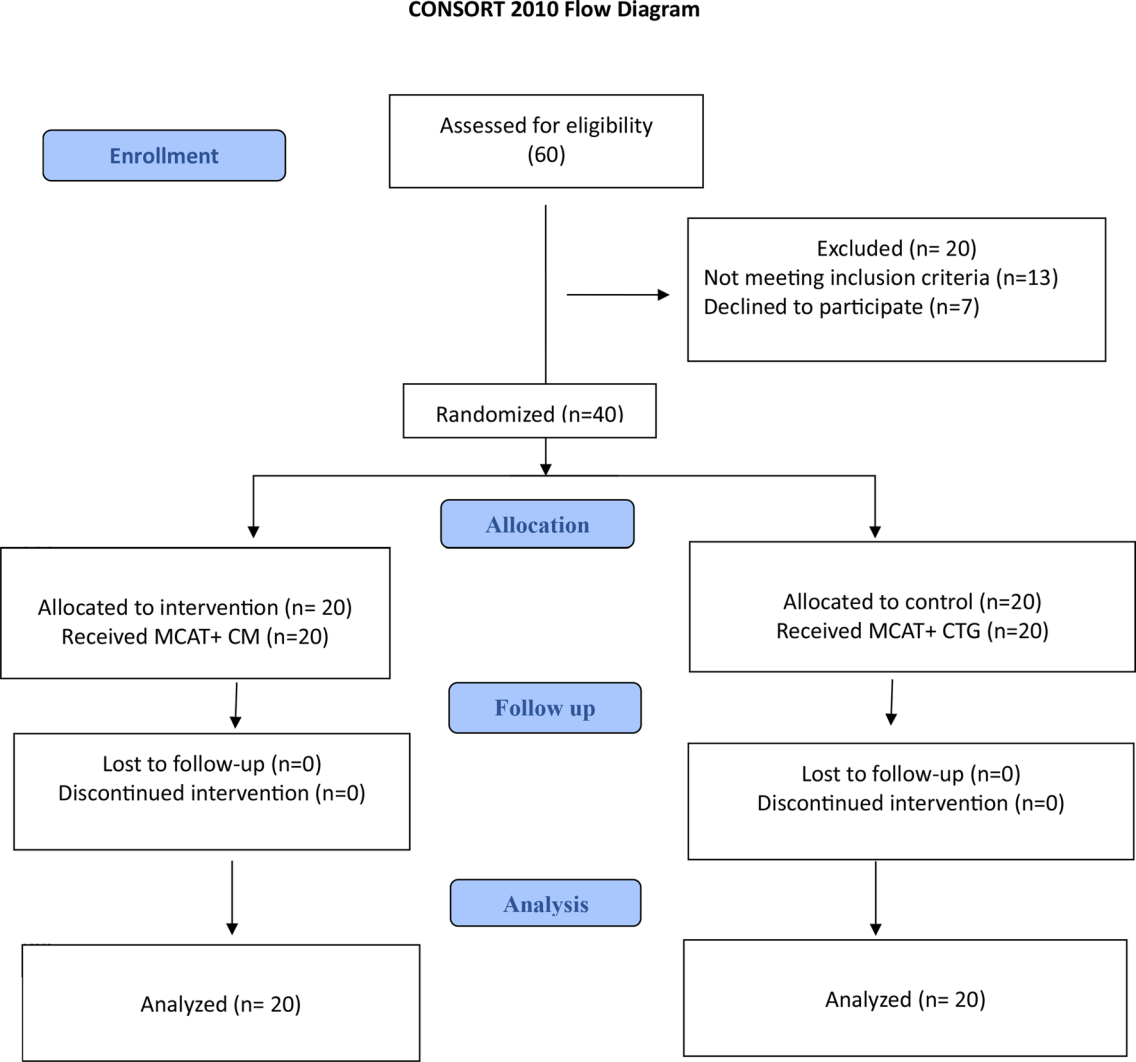
Consolidated Standards of Reporting Trials (CONSORT) flow chart for trial recruitment

According to the eligibility criteria of our study, sixty patients were screened. The participants were evaluated according to the prespecified inclusion and exclusion criteria. After careful assessment, 20 patients were excluded from the study due to the following: 13 patients didn’t match the inclusion criteria while 7 patients declined to participate after discussing the study protocol with them. For the remaining 40 eligible patients, we followed the randomization process to ensure unbiased allocation to the study groups. Twenty patients were allocated to each group while all the participants committed to the study till the end (6 months). The details of enrollment are described in the consort flow diagram (Figure. 3).

**Consort 2010 Flow Diagram**.

### Outcome evaluations

The entire 40 patients enrolled in the study were randomized as 20 patients to either control or test group. All the participants accomplished the study (from July 2022 to February 2024) till the end of the follow-up period (3, and 6 months). The demographic data of the patients and their characteristics were summarized in Table 1.

**Table 1:** Demographic data of the participants in the control and study groups

Demographic data	MCAT + CTG (*n* = 20)		MCAT + MUCODERM (*n* = 20)		Test of Significance.	*p*
	**No.**	**%**	**No.**	**%**	χ^2^= 0.100	0.752
Sex						
Male	10	50.0	11	55.0		
Female	10	50.0	9	45.0		
Age (years)					t = 0.099	0.921
Mean ± SD.	30.85 ± 6.52		31.05 ± 6.23			
Median (Min.– Max.)	31.50 (21.0–44.0)		30.0 (22.0–44.0)			

SD: **Standard deviation t: Student t-test** χ^2^: **Chi square test** p: p value for comparing between the two studied groups

#### Primary outcome variables analysis AGW, RD, RW, MRC%

The values of the clinical parameters are presented in Tables 2 and 3; and 4. The primary outcome variables (AGW, RD, RW, MRC%) have improved from the baseline till the end of the study, representing a significant intragroup analysis through different follow-up periods (3, and 6 months) (p-value ≤ 0.05).

The AGW has improved significantly from the baseline to the end of the follow-up periods in both groups (p-value ≤ 0.05). The AGW increased from − 0.75 ± 1.12 to 1.90 ± 0.72 and from − 1.20 ± 0.71 to 1.65 ± 0.49 in the control group and test group respectively through the 6-month follow-up period. Intergroup analysis revealed no differences in AGW during all times of the assessment. Table 2.

Regarding the RD and RW, the intragroup analysis showed a statistically significant difference from the baseline to the final follow-up period in both groups (p-value ≤ 0.05). RD improved from 3.20 ± 0.70 to 0.10 ± 0.31, and from 3.85 ± 0.88 to 0.15 ± 0.37 in both control and test groups respectively. RW also decreased from 2.0 ± 0.56 to 0.10 ± 0.31, from 2.25 ± 0.64 to 0.30 ± 0.73 in both groups respectively. Although the CTG group had better clinical presentation than the CM group, the intergroup analysis did not show significant differences regarding RD and RW. Table 2.

Regarding the improvement in the MRC%, there was a significant improvement after 3 months and 6 months postoperatively in both groups. For the control group, the MRC% was 93.33 ± 11.98 after 3 months, which further improved to 97.08 ± 9.09 after 6 months. The test group’s results improved from 91.0 ± 13.14 after 3 months to an even better result of 96.75 ± 7.99 after 6 months. Intergroup analysis revealed no significant differences from the baseline to the final follow-up. Table 2.

**Table 2:** Comparison between the two studied groups according to primary outcome in each period

Primary outcomes	MCAT + CTG (n = 20)			MCAT + MUCODERM (n = 20)			(Inter group analysis)			Baseline to After 3M intra group analysis(p-value)		Baseline to After 6M intra group analysis(P-value)	
	Baseline	After 3M	After 6M	Baseline	After 3M	After 6M	p_1_	p_2_	p_3_	MCAT + CTG	MCAT + MUCODERM	MCAT + CTG	MCAT + MUCODERM
**Attached gingiva width (AGW)**	-0.75 ± 1.12	1.15 ± 0.37	1.90 ± 0.72	-1.20 ± 0.71	1.05 ± 0.39	1.65 ± 0.49	0.174	0.620	0.341	0.001^*^	< 0.001^*^	< 0.001^*^	< 0.001^*^
**Recession depth (RD)**	3.20 ± 0.70	0.25 ± 0.44	0.10 ± 0.31	3.85 ± 0.88	0.40 ± 0.60	0.15 ± 0.37	0.026^*^	0.547	0.799	< 0.001^*^	< 0.001^*^	< 0.001^*^	< 0.001^*^
**Recession width (RW)**	2.0 ± 0.56	0.25 ± 0.44	0.10 ± 0.31	2.25 ± 0.64	0.65 ± 0.93	0.30 ± 0.73	0.253	0.355	0.738	< 0.001^*^	0.001^*^	< 0.001^*^	< 0.001^*^
**Mean root coverage% (MRC%)**	0.0 ± 0.0	93.33 ± 11.98	97.08 ± 9.09	0.0 ± 0.0	91.0 ± 13.14	96.75 ± 7.99	1.000	0.678	0.841	< 0.001^*^	0.001^*^	< 0.001^*^	< 0.001^*^

Data was expressed using Mean ± SD. SD: **Standard deviation**p_1_: p value for **Mann Whitney test** for comparing between two groups **at baseline**p_2_: p value for **Mann Whitney test** for comparing between two groups **After 3 months**p_3_: p value for **Mann Whitney test** for comparing between two groups **After 6 months** *: Statistically significant at *p* ≤ 0.05

#### Secondary outcome variables analysis GT, KTW, CAL, PPD, PES, RES, PI, GI

The secondary outcome variables are presented in Table 3. In terms of GT, there was an average gain of 3.35 ± 0.49 mm for the CTG group and 2.80 ± 0.41 mm for the CM group with significant improvement from the baseline to the final follow-up. Comparing the two groups, the CTG group exhibited better than the CM group. The intergroup analysis was significant (*p* ≤ 0.05) after 6 months. Table 3.

Regarding the KTW, there was a significant improvement after the final follow-up period in both groups. The 6-month results were improved with an average gain of 3.15 ± 0.37 mm and 2.80 ± 0.41 mm for CTG and CM groups respectively. Regarding CAL and PPD there was a significant improvement after 6 months follow-up period (p-value ≤ 0.05). PES and RES were used to assess patient satisfaction with gingival recession treatment, their values were improved significantly in both groups after 6 months. The intergroup analysis of the secondary outcomes (KTW, CAL, PPD, PES, and RES) did not show significant differences after the 6-month final follow-up. Table 3.

**Table 3:** Comparison between the two studied groups according to secondary outcome in each period

	MCAT + CTG (*n* = 20)			MCAT + MUCODERM (*n* = 20)			(Inter group analysis)			Baseline to After 3 M intra group analysis(*p*-value)		Baseline to After 6 M intra group analysis(*P*-value)	
Secondary outcomes	Baseline	After 3 M	After 6 M	Baseline	After 3 M	After 6 M	*p*_1_	*p*_2_	*p*_3_	MCAT + CTG	MCAT + MUCODERM	MCAT + CTG	MCAT + MUCODERM
**Keratinized tissue width**	1.90 ± 0.85	3.15 ± 0.37	3.35 ± 0.49	1.50 ± 0.67	2.80 ± 0.41	3.0 ± 0.0	0.121	0.086	0.060	0.001^*^	< 0.001^*^	< 0.001^*^	< 0.001^*^
**Gingival thickness**	0.78 ± 0.30	2.90 ± 0.31	3.35 ± 0.49	0.93 ± 0.18	2.75 ± 0.44	2.80 ± 0.41	0.165	0.429	0.009^*^	< 0.001^*^	< 0.001^*^	< 0.001^*^	< 0.001^*^
**Clinical attachment level**	5.85 ± 0.99	2.25 ± 0.44	1.55 ± 0.51	6.55 ± 0.89	2.15 ± 0.88	1.50 ± 0.69	0.068	0.698	0.620	< 0.001^*^	< 0.001^*^	< 0.001^*^	< 0.001^*^
**Pink esthetic score**	8.55 ± 1.32	13.75 ± 0.44	13.90 ± 0.31	9.05 ± 1.64	13.65 ± 0.49	13.90 ± 0.31	0.583	0.602	1.000	< 0.001^*^	< 0.001^*^	< 0.001^*^	< 0.001^*^
**Root coverage esthetic score**	2.10 ± 0.72	9.25 ± 1.33	9.70 ± 0.92	1.45 ± 0.51	8.95 ± 1.47	9.70 ± 0.92	0.008^*^	0.602	1.000	< 0.001^*^	< 0.001^*^	< 0.001^*^	< 0.001^*^
**Patient satisfaction PROM (VAS)**	0.0 ± 0.0	9.0 ± 0.56	9.40 ± 0.60	0.0 ± 0.0	8.45 ± 0.83	9.10 ± 0.55	1.000	0.076	0.157	< 0.001^*^	< 0.001^*^	< 0.001^*^	< 0.001^*^
**Plaque index**	0.25 ± 0.44	0.0 ± 0.0	0.0 ± 0.0	0.30 ± 0.47	0.0 ± 0.0	0.0 ± 0.0	0.799	1.000	1.000				
**Gingival index**	0.0 ± 0.0	0.10 ± 0.31	0.0 ± 0.0	0.0 ± 0.0	0.15 ± 0.37	0.0 ± 0.0	1.000	0.799	1.000				
**Periodontal Probing depth (mm)**	2.65 ± 0.59	2.0 ± 0.0	1.45 ± 0.51	2.70 ± 0.47	1.75 ± 0.44	1.35 ± 0.49	0.947	0.183	0.602	0.011^*^	< 0.001^*^	0.001^*^	< 0.001^*^

Data was expressed using Mean ± SD. SD: **Standard deviation** p_1_: p value for **Mann Whitney test** for comparing between two groups **at baseline** p_2_: p value for **Mann Whitney test** for comparing between two groups **After 3 months** p_3_: p value for **Mann Whitney test** for comparing between two groups **After 6 months** *: Statistically significant at *p* ≤ 0.05

Regarding the patient’s perception of the post-operative pain after the surgical procedures, VAS measures from the baseline to 2 weeks postoperative were assessed. There was no significant difference between the two surgical techniques after 2 weeks. However, the CTG group demonstrated significant differences during the early days of healing, with patients reporting greater pain and discomfort compared to the CM group. Table 4.

**Table 4:** Comparison of the visual analogue scale (VAS) in each group between the three study periods

	visual analogue scale (VAS)				Fr		*p*
	Baseline (1st day after surgery)	after 1 week	After 2 weeks				
MCAT + CTG (*n* = 20)							
Mean ± SD.	4.30 ± 0.47	1.0 ± 0.0		0.0 ± 0.0		40.00^*^	< 0.001^*^
Median (Min.– Max.)	4.0 (4.0–5.0)	1.0 (1.0–1.0)		0.0 (0.0–0.0)			
Significance between periods	p_1_ = 0.002^*^, p_2_ < 0.001^*^, p_3_ = 0.002^*^						
MCAT + MUCODERM (*n* = 20)							
Mean ± SD.	4.15 ± 0.37	1.10 ± 0.31		0.10 ± 0.31		40.00^*^	< 0.001^*^
Median (Min.– Max.)	4.0 (4.0–5.0)	1.0 (1.0–2.0)		0.0 (0.0–1.0)			
Significance between periods.	p_1_ = 0.002^*^, p_2_ < 0.001^*^, p_3_ = 0.002^*^						

SD: **Standard deviation Fr**: **Friedman test**, Sig. bet. Periods was done using **Post Hoc Test** (**Dunn’s)** p: p value for comparing between the three studied periods in each group p_1_: p value for comparing between **Baseline** and **after 1 week** p_2_: p value for comparing between **Baseline** and **After 2 weeks**p_3_: p value for comparing between **After 1 week** and **After 2 weeks** *: Statistically significant at *p* ≤ 0.05

## Discussion

CAF with CTG was the gold standard technique for gingival recession treatment for many years [40]. Despite this, it required complex flap designs featuring multiple layers of varying thickness [41]. Therefore, alternative techniques and materials were used for gingival recession coverage with predictable and stable results [42]. This was in agreement with Aroca et al. who proposed and assessed the outcomes of the MCAT for gingival recession treatment [21]. Also, the outcomes were in agreement with the Tavelli et al. systematic review, which assessed the localized and multiple gingival recession management using the tunnel technique. This makes the combination of tunneling technique and grafting material crucial in treating gingival recession [43–45]. 

The primary and secondary outcomes were significantly improved in both groups after the final follow-up period when compared to the baseline. The marked improvement in the clinical outcomes (GT) belonged to the control group (MCAT + CTG). This result was in agreement with the results of Chambrone et al. systematic review which compared the use of CTG and CM for single and multiple gingival recession treatment using tunneling techniques [44], also the findings were correlated with the Dia et al. study in 2019, that analyzed the outcomes of isolated gingival recession treatment [28]. 

In our study we noticed a gain in the AGW in both groups. This was correlated with Tarnow et al. study who adopted the new definition of attached gingiva that differentiates between AGW and KTW [5]. The explanation of this gain in AGW may be related to the tunneling design of the MCAT, which preserves the pristine attachment on the non-exposed root surface which acts later as a reservoir of cell adhesion for new attachment after recession treatment. The fact that MCAT does not include a flap elevation may be another cause of such gain in the AGW, due to the exclusion of the possibility of bone loss which is commonly noted in the literature as a drawback associated with flap surgery [46]. 

After 6 months this RCT revealed significant improvement in terms of RD, and RW compared to baseline in both groups. This finding was correlated with the results of Monlar et al. who conducted a clinical trial to assess the 9-year follow-up of multiple adjacent RT1 gingival recession after treatment using the tunneling technique [47]. The possible explanation of this may be attributed to the tunnelling technique itself and due to the creeping phenomenon that occurred with the soft tissue grafting procedures as a spontaneous coronal migration of the gingival margin, that may be attributed to continuous fibroblast activity promoting tissue migration and attachment. This is consistent with the results of a systematic review that revealed postsurgical migration of the gingival margin after recession treatment [48]. 

The MRC % had improved after 6 months of treatment with a value of 97.08 ± 9.09%, and 96.75 ± 7.99% for the control and the test group respectively. This improved MRC% may be attributed to the efficacy of the MCAT in displacing the gingival margin in a coronal position to the level of the CEJ without any tension from the muscle on the displaced gingival margin, also due to the creeping phenomenon that is usually observed with CTG. In contrast to the CM, which is less mature and needs time for graft incorporation and vascularization. Our results agreed with Pietruska et al. who achieved similar MRC% with CTG and CM for Miller class I and II gingival recession and with Rakasevic et al. who used both CTG and CM for multiple gingival recession treatment [49, 50]. 

MCAT + CTG treated sites showed better improvement than MCAT + CM from baseline to 6 months regarding the GT, while the remaining secondary outcome variables (KTW, CAL gain, and PPD reduction) were the same in both groups. One of the possible explanations for this is the genetically predetermined nature of the CTG, which is more mature than the CM. Also, the tunneling technique itself, which is employed in both groups, acts as a booster effect that may enhance the cell attachment, as it provides the mature CTG with immediate blood supply making the graft cells more productive and richer in connective tissue collagen fibers. Our results agreed with Zuhr et al. who noted that the flap design in this technique improves the vascular supply to the CTG, ensuring precise vascularization essential for graft incorporation. This enhanced vascularization also contributes to the long-term survival of the CTG [18, 51]. Another explanation for this; is the tendency for fibrotic response in CTG making the treated site liable to thicken over time with a resultant gain in thickness. The explanation for this may be due to the biological nature of the graft materials used, the CTG is more mature compared to the CM due to its autogenous nature including the presence of fibroblasts and growth factors that enhance tissue integration and vascularization. In contrast, the CM is less mature and may attributed to volume loss if exposed during healing, as it acts primarily as a scaffold, facilitating tissue regeneration by promoting cell attachment, migration, and neovascularization, but lacking the inherent biological activity of autogenous grafts. This is correlated with the study of Dellavia et, al who assessed the human palatal donor site for ridge augmentation [52]. 

Regarding the root coverage aesthetic outcomes perception, VAS and PROMs were used to evaluate patient satisfaction with the treatment and its outcomes [29, 30]. In terms of patient satisfaction and perception of the treatment outcomes, as assessed by using PES and RES as professional-centered aesthetic parameters, both groups had improved results. These methods are widely used to measure aesthetic outcomes after surgical intervention. The final PES and RES values were similar among both groups in the present study after 6 months. Although the control group had better tissue texture and fewer scars, the difference did not last for a long time throughout the follow-up, where both groups showed similar tissue aesthetics over the long-term follow-up. These findings: indicate that, after 6 months of follow-up the stability of aesthetic outcomes, is the same as in many other studies such as Sculean et al., who assessed the esthetic outcome after treatment using the MCAT technique [31, 51]. Also, both groups were reported to have MGJ re-alignment harmoniously after treatment. This could be related to the effects of the tunneling technique, which prevents the distortion of the MGJ, unlike CAF which uses the vertical releasing incisions in the flap design. This explanation is supported by the results of Kerner et al., who evaluated the qualitative cosmetic outcome after gingival recession treatment [53]. 

There was no patient loss during follow-up in both groups as is obvious from the baseline to 6 months because these results may have a significant effect on the outcomes. Notably, the present study had no attrition rate compared to other studies, when the sample sizes and follow-up timing were similar [54]. Additionally, the clinical outcomes were assessed by the same blinded investigator throughout all study follow-up periods, enhancing the accuracy of the findings.

### Conclusion

Both study groups provided comparable and stable clinical outcomes in which both materials can be used with satisfactory outcomes while CM may be used as an alternative for CTG for gingival recession treatment. This RCT provides clinical evidence that the MCAT technique can be effectively utilized in mucogingival surgeries, improving soft tissue stability, esthetic outcomes, and patient’s quality of life in a minimally invasive approach.

### Limitations

According to the design of this clinical trial, there are limitations reported. Specifically, this RCT has focused on isolated recession defect (RT1) which may limit the observed results regarding all other types of gingival recession defects. In addition, we report a small sample size and short follow-up period in the current study. So, we recommend future prospective studies with larger sample sizes and longer follow-up periods. Also, multicenter clinical trials are recommended for technique validity. Moreover, further research is needed to assess the role of surgeon skill on the treatment outcomes for better evidence of the impact of the different factors, techniques, and materials on the outcomes of treatment of different classes of gingival recession.

## Data Availability

The corresponding author can provide the datasets produced and/or examined during this study upon request to be accessed by the public.
